# Supplementary material for: Lived experience of nutrition impact symptoms among patients undergoing chemotherapy in Ethiopia: An interpretative phenomenological analysis
Source: PLoS One. 2025 Nov 14;20(11):e0337040. doi: 10.1371/journal.pone.0337040 (PMC12617936; doi:10.1371/journal.pone.0337040)
Supplement: S1 Table — (DOCX) [file pone.0337040.s001.docx]

**S2 file. Standards for Reporting Qualitative Research (SRQR) Checklist**

| **No** | **Topic** | **Location (page & line no)** |
| --- | --- | --- |
|  | Title and abstract |  |
| S1 | Tile | Page 1 |
| S2 | Abstract | Page 2 |
|  | **Introduction** |  |
| S3 | Problem formulation | Page 3, lines 71-73, and page - 4, lines 101-108 |
| S4 | Purpose or research question | Page 4, line 108 & 109. |
|  | **Methods** |  |
| S5 | Qualitative approach and research paradigm | Page 4, lines 94 - 100 |
| S6 | Researcher characteristics and reflexivity | Page 6, lines 147 - 163 |
| S7 | Context | Page 5, lines 116 - 125 |
| S8 | Sampling strategy | Page 5, lines 127 - 137 |
| S9 | Ethical issues pertaining to human subjects | Page 12, 266 - 273 |
| S10 | Data collection methods | Page 7, lines 176 - 179 |
| S11 | Data collection instruments and technologies | Page 8, lines 194 - 195 |
| S12 | Units of study | Page 5, lines 127 - 128 |
| S13 | Data processing | Page 9, lines 204 - 212 |
| S14 | Data analysis | Page 9, lines 216 - 229 |
| S15 | Techniques to enhance trustworthiness | Page 11, lines 235 - 265 |
|  | **Results/findings** |  |
| S16 | Synthesis and interpretation | Page 13, lines 281 – 292 and  Page 18, lines 408 – 412, Figure 3. |
| S17 | Links to empirical data | Page 13– 18, lines 293 - 407 |
|  | **Discussion** |  |
| S18 | Integration with prior work, implications, transferability, and contribution(s) to the field | Page 18 - 23, lines 413 - 562 |
| S19 | Limitations | Page 23, lines 563 - 571 |
|  | **Other** |  |
| S20 | Conflicts of interest | Page 24, line 596 |
| S21 | Funding | Page 24, line 594 |
