# Supplementary material for: Lived experience of nutrition impact symptoms among patients undergoing chemotherapy in Ethiopia: An interpretative phenomenological analysis
Source: PLoS One. 2025 Nov 14;20(11):e0337040. doi: 10.1371/journal.pone.0337040 (PMC12617936; doi:10.1371/journal.pone.0337040)
Supplement: Attachment — Submitted filename: Comment_One.docx [file pone.0337040.s003.docx]

**Dear Academic Editor,**

I hope this message finds you well. I am writing to provide my comments on the manuscript titled "[Lived Experience of Nutrition Impact Symptoms among Patients Undergoing Chemotherapy in Ethiopia: An Interpretative Phenomenological study]" (Manuscript ID: [PONE-D-25-19003]) that I had the opportunity to review.

I appreciate the opportunity to review this manuscript and hope that my feedback will be helpful to the authors in improving their work. Please let me know if you require any further information or clarification regarding my comments.

Thank you for considering my review

Overall, I found the manuscript to be well-structured and innovative. However, I have some suggestions and comments that I believe could enhance the quality of the work, which are presented below.

**Comment #1:** The title stated in lines no 1 and 2 stated that “Lived Experience of Nutrition Impact Symptoms among Patients Undergoing Chemotherapy in Ethiopia…” But the concept that you stated in the abstract part in the background section, lines no 32 and 33, is “This study aimed to explore the lived experiences of symptoms that hinder food intake among chemotherapy recipients at a major cancer center in Ethiopia.” Hence, there is a misalignment between the title and the objective. Please revisit it.

**Comment #2:** In the abstract in the method section, line no 35, you mentioned “…purposive sampling…” This sampling method is the most preferred in qualitative studies, but it has many types of sampling techniques. Please state the specific type of purposive sampling you used in your study.

**Comment #3:** In the abstract in the method section at line no 35, you stated “… involving 26…” You did not mention how you reached at this level. Please precisely mention such size determination method that you applied.

**Comment #4:** In the abstract in the method section at lines no 36 to 37, you stated “…. transcribed verbatim in Amharic, and then translated into English”. You used a verbatim approach for transcription, but you did not mention the method that you used for translation, and you also missed it in the method part. Please precisely include this approach here.

Comment #5: The gaps that you what to fill in this study using a qualitative approach, including the paradigm, are sufficiently described. Please include it.

**Comment #6:** In the methods part in the study participants and selection section at lines no 115 to 117, you stated “….A total of 26 participants were purposively selected based on their experiences with nutrition-impact symptoms, diverse age groups, sexes, cancer types, stages, and treatment regimen…” This indicated that diverse criteria were used to recruit the participants. Hence, it is necessary to indicate the types of purposive sampling you used.

**Comment #7:** In the methods section from line no 120 to 132, you describe the Reflexivity statement and bracketing.” It is good to understand the position of the researchers about the issue that was investigated and how researchers' bias is introduced into the findings. But only the first author's and one other author's issues related to the topic were described. Hence, it lacks the position of other authors on the issue. Disclosure about the presence of cancer in the families of the authors is essential. Hence, precisely include such and other related events.

**Comment #8:** In the methods part in the data collection section, from lines no 142 to 146, you describe how you interview the participants. In this part, it is also necessary to include the mechanisms you used to maintain and engage the interest of the participants during the interview time.

**Comments #9:** In cases where the audio quality was poor or the discussion required clarification, what mechanism did you apply during audio transcription and translation?

**Comment #10:** In the methods part, you described trustworthiness and rigor assurance mechanisms. It is good to include additional mechanisms that you applied to assure the trustworthiness of your findings (like an iterative process, modifications of study procedure in response to evolving study findings not adequately mentioned, figuring out contradicting ideas, and further verifying them, etc).

**Comment #11:** The discussion is supported by evidence, but some areas focus heavily on summarizing findings without giving meaning and specification of implications. Therefore, consider elaborating on how the findings challenge or confirm existing theories. **This is my major concern, please revisit this in-depth.**

**Comments # 12**: Please follow the qualitative research guideline of Plos one.
