# Supplementary material for: Lived experience of nutrition impact symptoms among patients undergoing chemotherapy in Ethiopia: An interpretative phenomenological analysis
Source: PLoS One. 2025 Nov 14;20(11):e0337040. doi: 10.1371/journal.pone.0337040 (PMC12617936; doi:10.1371/journal.pone.0337040)
Supplement: Attachment — Submitted filename: Reviewer Feedback_lived experience of NIS_Ethiopia.docx [file pone.0337040.s004.docx]

**Reviewer Feedback**

**Overall Assessment**

This is a well-conceived and timely qualitative study exploring the lived experiences of nutrition-impact symptoms (NIS) among Ethiopian cancer patients undergoing chemotherapy. The use of Interpretative Phenomenological Analysis (IPA) provides rich insights into patient perspectives.

However, there are areas where clarity, depth, and alignment with reporting standards could be strengthened.

**Comments/ suggestions:**

- **Abstract:** Well-written, but maybe include some more details in the results instead of just listing the themes and subthemes, giving some information on what each theme represents.
- **Introduction:**
  - *Line 69-72: “In our baseline cross-sectional study, we found that 61.7% of patients in major cancer centers in Ethiopia had at least one NIS before chemotherapy initiation, with expected increases during and after treatment (unpublished data)”*. I suggest using published information to support your argument.
  - *Line 74-75: you mention there are “Several qualitative studies have reported that NIS in individuals with cancer receiving treatments is associated with weight loss, poor quality of life, and decreased survival”* – but no reference, include references.
  - *Line 87-88: “However, the findings are limited to British and American contexts, making them less applicable to other cultural settings with different dietary habits”*- by this, are we assuming the symptoms vary by setting or by dietary habits? It is good to clarify
  - *Line 89-90: “closer examination of previously published studies lacks a comprehensive understanding of patients' experiences with NIS”-* clearly indicate what they are missing and what this study is doing differently to fill the gaps.
- **Terminology:** Use consistent phrasing for “nutrition-impact symptoms” (sometimes abbreviated, sometimes not).
- **Methodology**
  - Clarify how “information saturation” was determined—was it based on thematic redundancy or coding stability?
  - **Participant selection**: of the patients receiving chemo; it is not clear how the 26 were selected. How did you approach the patients? Who approached them? What criteria did you use to include them in the study? Did you have any exclusion criteria?
  - **Reflexivity section:**
    - I think in this section, you should include whether you are currently working at the center? are you also the nutritionist currently providing care for the patients?
    - In addition to taking the course, do you have any experience with qualitative research, or does anyone in your research group have?
  - ***Bracketing*** *Line 128-129: “the principal investigator’s a priori perspectives and preconceived ideas about nutrition-impact symptoms were identified and documented before starting data collection and analysis”-* What do you mean? How was it documented? And how was it reflected upon during analysis? Give details
  - **Data collection:**
    - *Line 137-138-* who did the back translation and how was the semantic equivalence checked?
    - *Line 142-143:* were the interviews conducted before or after their visit to their physician
  - **Data Analysis:**
    - *Line 148*: Was the analysis only conducted by the primary author? Was there any process to verify the codes? Themes? And final interpretation with any of the research team?
    - *Line 150- “To minimize potential bias, the researcher employed bracketing during the analysis process”*- what do you mean? What was the process, and how did it minimize bias
  - **Trustworthiness and rigor:**
    - *Line 170-171:* What do you mean by the researcher included reflexive statements to provide context for interpreting the findings?
    - *Line 172: How was the cross-validation process? What happened when there were no agreements between the two students? What was done when there were differences in coding and interpretative?*
    - *Line 173: who was debriefed and what do you mean consensus was reached? among which group?*
- **Results:**
- General Comments: you have included too many quotes. I suggest selecting a maximum of one or two and removing the others. Alternatively, if you believe all the quotes are relevant, you could provide a brief description before each one to highlight the new concepts or ideas that each quote supports.
  - *Line 202:* The description of how you achieved the results should be moved to the methods section.
  - *Line 205-211: Instead of mentioning the number of sub-themes under each theme, I suggest briefly highlighting the three themes that emerged and what each represents.*
  - *Line 220-221-* You mean to say the participants compared it early pregnancy symptoms?
  - *Line 228- when using the quotes, it is good to include some description, for example, (male, age 70). It is good if you can do that for all quotes; it makes it easier to relate to what is being said.*
  - *Line 248-250: It is unusual to include definitions in the results section. Consider relocating other sections if necessary*
  - *For category 1.1-1.2: both are focusing treatment related symptoms: physical and psychological. I suggest considering merging them and removing sub-themes*
  - *For category 1.3 it is different from the rest, and I suggest you put it under a separate theme highlighting the different coping mechanisms. For example, you could discuss the use of pharmacological, dietary, and economic coping strategies. It would be helpful to expand on each of these and include relevant quotes. Additionally, I believe that Theme 2 can be integrated with this, as individual tolerance to different foods and discovering what works best for each person is a crucial aspect of coping strategies.*
  - *Line 286: 291: Are you referring to their preferences in general or preference and tolerance while taking chemo?*
  - Line 308-311: Doesn’t this reflect a lack of specialized nutritional support in general, instead of a misconception?
