# Supplementary material for: Lived experience of nutrition impact symptoms among patients undergoing chemotherapy in Ethiopia: An interpretative phenomenological analysis
Source: PLoS One. 2025 Nov 14;20(11):e0337040. doi: 10.1371/journal.pone.0337040 (PMC12617936; doi:10.1371/journal.pone.0337040)
Supplement: Attachment — Submitted filename: Response to Reviewers.docx [file pone.0337040.s005.docx]

**Response to the Editor and Reviewers**

Firstly, on behalf of the research team, I would like to thank the editor and reviewers for their valuable time and constructive comments on our manuscript. Their feedback will enhance the scientific rigor of the work. Below, I provide detailed responses to each comment. All modifications are highlighted in red color with the “revised manuscript with track changes”.

**Response to the editor**

1. A response to reviewers, a revised manuscript with track changes, and a clean version of the manuscript are uploaded.
2. The manuscript has been prepared in accordance with PLOS ONE's style requirements. Its organization has been verified using the Standards for Reporting Qualitative Research (SRQR) checklist, as recommended by the journal. The checklist is also uploaded to the submission system as supporting information.
3. The data sharing statement has been revised to: “All relevant data are within the manuscript and its supporting information files.” The dataset is exported directly from MAXQDA software to an Excel sheet, which is uploaded as a supporting information file. I believe the Excel format is clear and straightforward for potential replication.
4. Dr. Diana Cardenas considers her contribution limited to being recognized as a co-author and requests her name to be included in the acknowledgment section. Consequently, her name has been moved to the acknowledgment. I apologize for any inconvenience this may have caused.
5. The reviewers did not recommend citing specific previously published works.

**Response to reviewer – 1**

**Comment #1:** The title stated in lines no 1 and 2 stated that “Lived Experience of Nutrition Impact Symptoms among Patients Undergoing Chemotherapy in Ethiopia…” But the concept that you stated in the abstract part in the background section, lines no 32 and 33, is “This study aimed to explore the lived experiences of symptoms that hinder food intake among chemotherapy recipients at a major cancer center in Ethiopia.” Hence, there is a misalignment between the title and the objective. Please revisit it.

**Response**: Accepted. Although, nutrition impact symptoms refers the symptoms that hinder food intake, the comment to use consistent terms throughout the manuscript is acceptable and incorporated. **Page 2, line** **31-32.**

**Comment #2:** In the abstract in the method section, line no 35, you mentioned “…purposive sampling…” This sampling method is the most preferred in qualitative studies, but it has many types of sampling techniques. Please state the specific type of purposive sampling you used in your study.

**Response**: Accepted. The specific sampling technique used in the study was a heterogeneous sampling (maximum variation sampling) technique where we mixed patients by gender, cancer type, treatment setting (outpatient versus inpatient), the type of therapeutic agents, and the number of chemotherapy cycles the patient was taking. The specific sampling technique is incorporated in both the abstract and method sections. **Page 2, lines 36** and **page 5, lines 132 - 137**, respectively.

**Comment #3:** In the abstract in the method section at line no 35, you stated “… involving 26…” You did not mention how you reached at this level. Please precisely mention such size determination method that you applied.

**Response**: Accepted. Notably, saturation in thematic analysis is a debated topic among qualitative researchers. In our study, the sample size was determined when no new information emerged during data collection (data saturation) and no new codes or themes appeared during analysis (thematic saturation). After completing data analysis with 26 samples, we collected two additional verification cases to capture any new information or codes that might arise. At this point, saturation was declared due to the absence of new responses from the interviews, and the data analysis confirmed existing codes rather than revealing new ones (thematic saturation). Consistently, the sample size in our study is somewhat larger than that of a systematic review, which indicated that saturation in homogeneous study populations (single country) is often reached within 9 to 17 interviews.

Based on the comment, a precise statement on how the sample size was determined using data and thematic saturation has been included in the abstract **(lines 35-36)**. Additionally, detailed descriptions of the procedures have been added to the methods section on **page 6, lines 138-146**.

**Comment #4:** In the abstract in the method section at lines no 36 to 37, you stated “…. transcribed verbatim in Amharic, and then translated into English”. You used a verbatim approach for transcription, but you did not mention the method that you used for translation, and you also missed it in the method part. Please precisely include this approach here.

**Response:** Accepted. As this study is an interpretative phenomenological analysis, we utilized contextual translation. This approach is essential because the wording, as well as the patients' disease status and socio-economic context, significantly contribute to the interpretation of their symptom experiences. Contextual translation preserves the intended message and socio-cultural nuances. The type of translation is mentioned in the abstract **(line 38)** and presented in detail in the methods section (**Page 9, lines 210-212).**

**Comment #5:** The gaps that you what to fill in this study using a qualitative approach, including the paradigm, are sufficiently described. Please include it.

**Response:** I take this comment as “not sufficiently described.” The justification for the qualitative approach and constructivist paradigm of this study is detailed on **page 4, lines 94-100.**

**Comment #6:** In the methods part in the study participants and selection section at lines no 115 to 117, you stated “….A total of 26 participants were purposively selected based on their experiences with nutrition-impact symptoms, diverse age groups, sexes, cancer types, stages, and treatment regimen…” This indicated that diverse criteria were used to recruit the participants. Hence, it is necessary to indicate the types of purposive sampling you used.

**Response:** Accepted. This question is addressed in comment #2. The heterogeneous sampling technique defines the specific method we used in our study. Detailed information about this sampling technique can be found on **page 5, lines 132–137.**

**Comment #7:** In the methods section from line no 120 to 132, you describe the Reflexivity statement and bracketing.” It is good to understand the position of the researchers about the issue that was investigated and how researchers' bias is introduced into the findings. But only the first author's and one other author's issues related to the topic were described. Hence, it lacks the position of other authors on the issue. Disclosure about the presence of cancer in the families of the authors is essential. Hence, precisely include such and other related events.

**Response:** Accepted. In this section, the second reviewer also suggested adding the professional background and previous experience in qualitative research of the principal investigator and co-authors. A statement detailing this professional and qualitative research experience is included in the reflexive statement on **page 6, lines 148-163.**

**Comment #8:** In the methods part in the data collection section, from lines no 142 to 146, you describe how you interview the participants. In this part, it is also necessary to include the mechanisms you used to maintain and engage the interest of the participants during the interview time.

**Response**: Accepted. During the interview, distractions were minimized by turning off mobile phones, selecting quiet times in the wards, and conducting interviews for patients undergoing outpatient chemotherapy in a separate room (the head nurse’s office). Active listening, follow-up questions, maintaining neutrality regarding patients’ experiences, and adapting to emotional responses were used during the interview. Additional details can be found in the methods section on **page 8, lines 198-202.**

**Comments #9:** In cases where the audio quality was poor or the discussion required clarification, what mechanism did you apply during audio transcription and translation?

**Response:** During the transcription process, audio recordings of suboptimal quality were addressed by employing bracketed ellipses [...] and timestamp annotations to denote technical issues, such as [00:25:10 - background noise interference]. Additionally, segments of the transcript were flagged for subsequent verification with participants, either during their next chemotherapy cycle or via telephone communication if they were undergoing their final chemotherapy session at the time of the interview.

While not frequent, the inclusion of statements detailing procedures for addressing poor quality audio records, primarily background noise interference in the inpatient wards, is acceptable and has been incorporated into the data analysis section. **Page 9, lines 205 – 210.**

**Comment #10:** In the methods part, you described trustworthiness and rigor assurance mechanisms. It is good to include additional mechanisms that you applied to assure the trustworthiness of your findings (like an iterative process, modifications of study procedure in response to evolving study findings not adequately mentioned, figuring out contradicting ideas, and further verifying them, etc).

**Response: Accepted.** Familiarization with the content through an iterative process (repeated listening and reading) is reported in the methodology (**page 11, lines 238 - 242**). However, in this study, modification of the study procedure was not made in response to study findings. Importantly, the interview guide was pre-tested on three patients, resulting in the removal of redundant items and modifications to both the wording and the sequence of questions. The statement on pre-test of interview guide is stated in the methodology (**page 8, lines 187 - 189**).

Notably, unlike phenomenological studies, modifying study procedures based on ongoing analysis and findings is common in grounded theory and, to some extent, in ethnographic studies.

**Comment #10:** In the methods part, you described trustworthiness and rigor assurance mechanisms. It is good to include additional mechanisms that you applied to assure the trustworthiness of your findings (like an iterative process, modifications of study procedure in response to evolving study findings not adequately mentioned, figuring out contradicting ideas, and further verifying them, etc).

**Response:** Accepted. Some rigor assurance methods that were implemented but not detailed, such as prolonged engagement, immersion in data, and an audit trail, are now included in the trustworthiness section. Regarding dependability, in addition to peer debriefing with a doctoral student colleague, the overall study process, from the interview guide to manuscript preparation, was thoroughly evaluated by one of the co-authors (BSE), a qualitative research expert (Audit Trail). However, as explained above, the study procedure was not modified in response to evolving findings. I also understand that all the trustworthiness and rigor assurance mechanisms may not apply to all study designs and contexts. The expansion statement on trustworthiness and rigor assurance methods is included on **page 11, lines 238 – 241 and lines 251 – 254.**

Furthermore, during the coding process, we observed a contradiction in participants' food choices. Some preferred fruits and vegetables during chemotherapy, while others disliked them. Similarly, some used spices to boost appetite, whereas others avoided them due to stomach irritation. This lack of a clear pattern led to the development of the code "individualized food choice," which became an independent theme in the final analysis. We did not identify any additional contradictory findings.

**Comment #11:** The discussion is supported by evidence, but some areas focus heavily on summarizing findings without giving meaning and specification of implications. Therefore, consider elaborating on how the findings challenge or confirm existing theories. This is my major concern, please revisit this in-depth.

**Response**: Thank you for your comment. It is important to note that the discussion section is organized by sub-themes, with each paragraph addressing one sub-theme at a time. The final lines of each paragraph do not serve as summaries; rather, they highlight the potential implications of specific findings for improving clinical practice, which I believe is a fundamental component of a discussion section.

In response to this general comment, the discussion section has been thoroughly revised and expanded to contextualize the findings within the framework of other studies and existing theories. **See pages 18-23 or lines 420-569.**

**Comments # 12:** Please follow the qualitative research guideline of Plos one.

**Response**: The entire content is organized in accordance with the Standards for Reporting Qualitative Research (SRQR) checklist, which is one of the reporting guidelines recommended by the journal. As evidence, the filled checklist is uploaded as additional material in the submission system. Also, a statement indicating the reporting standard is added in the methods part **(Page 9, lines 229-231).**

**Response to reviewer – 2**

**Comment on the abstract:** Well-written, but maybe include some more details in the results instead of just listing the themes and subthemes, giving some information on what each theme represents.

**Response:** Accepted. Some details are added in the result section of the abstract, and the conclusion is also rephrased. **Lines 40 – 43.**

**Comment on the introduction: Line 69-72:** “In our baseline cross-sectional study, we found that 61.7% of patients in major cancer centers in Ethiopia had at least one NIS before chemotherapy initiation, with expected increases during and after treatment (unpublished data)”. I suggest using published information to support your argument.

**Response**: Accepted. At the time of submission of this manuscript, the cited manuscript was under review; now it is published and appropriately cited. **Reference number 16.**

**Comment on introduction:** Line 74-75: you mention there are “Several qualitative studies have reported that NIS in individuals with cancer receiving treatments is associated with weight loss, poor quality of life, and decreased survival” – but no reference, include references.

**Response:** It was assumed to serve as a topic sentence introducing a new paragraph. The studies referred to as "several" are listed and explained following this statement. However, if this creates confusion, I rephrased it to better function as a topic sentence for the new paragraph. **Page 3, lines 72 – 73.**

**Comment on introduction:** Line 87-88: “However, the findings are limited to British and American contexts, making them less applicable to other cultural settings with different dietary habits”- by this, are we assuming the symptoms vary by setting or by dietary habits? It is good to clarify.

**Response:** The trend of late cancer diagnosis in Ethiopia, where 80% of cases are identified at an advanced stage, coupled with a limited number of cancer and palliative care centers, ill-equipped oncology facilities, a shortage of nutritionists and dietitians, and economic barriers to accessing treatments and nutritious foods, is expected to increase the burden of nutrition-related symptoms in low-resource settings. The hypothesis is that while the disease remains consistent among countries, however, the environmental conditions may exacerbate the severity of these symptoms. Moreover, dietary habits vary across different settings. To enhance clarity, a summary of these rationales is provided in a commented paragraph. **Page 4, lines 86 - 90.**

**Comment on introduction:** Line 89-90: “closer examination of previously published studies lacks a comprehensive understanding of patients' experiences with NIS”- clearly indicate what they are missing and what this study is doing differently to fill the gaps.

**Response:** Accepted. The gap in previous studies conducted in Ethiopia and other contexts is clearly articulated. **See page 4, lines 99 – 113.**

**Comment:** Terminology: Use consistent phrasing for “nutrition-impact symptoms” (sometimes abbreviated, sometimes not).

**Response:** Accepted. To simplify understanding, I spelled out all the abbreviations of NIS throughout the manuscript.

**Comment on methodology:** Clarify how “information saturation” was determined—was it based on thematic redundancy or coding stability?

**Response**: This concern has also been raised by the first reviewer. While saturation in thematic analysis is a topic of debate among qualitative researchers, in this study, it was determined based on both data and thematic saturation. A precise statement regarding saturation is included in the abstract, and a detailed description of how it was achieved is presented in the methods section **(Page 6, lines 137-145).**

**Comment:** Participant selection: of the patients receiving chemo; it is not clear how the 26 were selected. How did you approach the patients? Who approached them? What criteria did you use to include them in the study? Did you have any exclusion criteria?

**Response:** The inclusion criteria consisted of adults aged 18 years or older who were diagnosed with solid tumors, undergoing active treatment, and had completed a minimum of two chemotherapy cycles. Additionally, participants needed to experience nutrition impact symptoms, be able to articulate their experiences, and voluntarily consent to participate. The principal investigator, with the assistance of a staff nurse from the cancer center, conducted the selection of participants. Further details regarding the recruitment criteria can be found in the methods section, specifically under "study participants and selection methods" **on pages 5, lines 126 – 136.**

**Comment on reflexivity section:** I think in this section, you should include whether you are currently working at the center? are you also the nutritionist currently providing care for the patients?

**Response:** I am a doctoral student in clinical nutrition at Addis Ababa University, with a background in clinical nursing and public health nutrition. Currently, I am not working in the study hospital. My exposure to cancer patients experiencing nutrition impact symptoms has been minimal, primarily in hospitals located in other regions of the country. This experience has helped me formulate the qualitative research question, which is part of my PhD thesis. In response to this comment, I have appended additional profiles of the principal investigator in the reflexive statement. **Page 6, lines 147 – 152.**

**Comment:** In addition to taking the course, do you have any experience with qualitative research, or does anyone in your research group have?

**Response:** The PI has not previously published a qualitative study. However, the qualitative research methods course included a group project that covered everything from topic selection to journal submission.

The co-authors of this study possess qualitative research experience. Among the five co-authors, four have contributed to qualitative research publications. Additionally, BSE is a public health nutritionist with extensive qualitative research expertise and has authored numerous publications, as well as secured grants. He thoroughly evaluated the overall process of this study (audit trail) to ensure trustworthiness and rigor. Further details regarding this aspect are included in the reflexive statement. **Page 6, lines 153 – 156.**

**Comment:** Bracketing Line 128-129: “the principal investigator’s a priori perspectives and preconceived ideas about nutrition-impact symptoms were identified and documented before starting data collection and analysis”- What do you mean? How was it documented? And how was it reflected upon during analysis? Give details

**Response:** Bracketing (epoché) is preventing researcher’s perspectives and preconceptions from influencing the qualitative analysis process particularly in phenomenological studies.

In this study, the concept of bracketing involves the PI explicitly recognizing his preexisting beliefs regarding nutrition-related symptoms, which are informed by prior clinical experience in a different region of the country. These personal perspectives and preconceived notions about nutritional symptoms are systematically brainstormed and recorded in a reflexive journal (notebook), primarily before starting data analysis. During the analysis phase, this journal is frequently reviewed to maintain an open mental state and to ensure that the findings accurately reflect the participants' experiences. The statement on bracketing is expanded and can be found on **page 9, lines 210 - 211.** Additionally, the detail description of the bracketing process used in the study is presented in methods part under the heading “bracketing” on **Page 7, lines** **162 – 172.**

**Comment on data collection.** Line 137-138- who did the back translation and how was the semantic equivalence checked?

**Response:** The interview guide was initially developed in English and subsequently translated into Amharic by the PI. It was then back-translated into English by a doctoral colleague proficient in both languages. This process ensured the preservation of meaning, cultural equivalence, and contextual accuracy of the question items and probes. Any discrepancies identified in some items were resolved through discussion. Detailed descriptions of the translation process can be found in the data collection section on **page 8, lines 177–182.**

**Comment on data collection:** Line 142-143: were the interviews conducted before or after their visit to their physician

**Response:** Yes. The interview was conducted after the physician's visits, and patients were determined as fit for chemotherapy. Fitness for chemotherapy is assessed every time patients come for treatment on their date of appointment. For patients taking outpatient chemotherapy, an interview was conducted after completing their treatment, bringing them to the head nurse's office for better environmental comfort. For the hospitalized patients, it was conducted in the afternoon after letting the attendants outside the ward. Most hospitalized patients were fatigued, making it impractical to relocate them to a separate room for the interview. These patients often wait 3 to 5 days in the hospital to complete treatment. Therefore, all the interviews were made after their physician visit and were deemed fit for chemotherapy. Some detailed information on the interview process is added in the data collection part on **page 8, lines 187 - 192.**

**Comment on data analysis:** Line 148: Was the analysis only conducted by the primary author? Was there any process to verify the codes? Themes? And final interpretation with any of the research team?

**Response:** Yes. This point is mentioned in detail in the trustworthiness part. Therefore, to ensure the trustworthiness of the findings, the transcript was independently coded by both the principal investigator and a doctoral student. Furthermore, an audit trail of the entire research process, including the coding procedures, was verified by one of the co-authors (BSE), who is an expert in qualitative research. The detailed statement on coding procedure is added under the data analysis section on **page 9, lines 221 – 223.**

**Comment on data analysis:** Line 150- “To minimize potential bias, the researcher employed bracketing during the analysis process”- what do you mean? What was the process, and how did it minimize bias.

**Response:** Thank you for the repeated concern about bracketing of preconceived perspectives. The overall issue related to bracketing has already been discussed above. Explaining the meaning and process of bracketing here may be redundant. I respectfully request the reviewer to look at the details presented under the heading “bracketing” on **Page 7, lines** **162 – 172.**

**Comment on trustworthiness and rigor:** Line 170-171: What do you mean by the researcher included reflexive statements to provide context for interpreting the findings?

**Response:** Reflexive statements are written declarations that document the researcher's potential biases, assumptions, and personal connections to the research. In this study, the Principal Investigator (PI), a doctoral student, conducts a qualitative study as part of his PhD thesis, which employs a mixed-methods design. The PI's experience supervising nursing students in cancer centers and completing a qualitative research methods course also informs these reflexive statements. Similarly, the profiles of coauthors and their relationships to the topic are transparently presented. The main purpose of these statements is to help readers understand the findings in the context of the researchers' backgrounds. Some qualitative researchers refer to this as a "researcher's position statement" to establish transparency regarding their relationship to the research topic. The information is detailed under the heading "reflexive statement" in the methods section on **page 6, lines 146 - 161.**

**Comment on trustworthiness and rigor:** Line 172: How was the cross-validation process? What happened when there were no agreements between the two students? What was done when there were differences in coding and interpretative? Line 173: who was debriefed and what do you mean consensus was reached? among which group?

**Response:** Two doctoral students independently coded the transcripts. The codes were then systematically compared by transcript sections. Consensus was reached for most codes, although some discrepancies in word choice were noted. In cases of disagreement, a discussion meeting was held where each coder explained their rationale and collaboratively reviewed the raw data. If disagreements persisted, one of the co-authors (BSE), a senior qualitative researcher, was consulted for resolution. These responses also clarify the debriefing process. For further detail, the description of the inter-coder process is **on page 11, lines 243 - 253.**

**Comment on result:** General Comments: you have included too many quotes. I suggest selecting a maximum of one or two and removing the others. Alternatively, if you believe all the quotes are relevant, you could provide a brief description before each one to highlight the new concepts or ideas that each quote supports.

**Response: Accepted.** The quotes have been minimized, and I have retained a few that I felt were essential, preceded by a description (**page 14 and page 15).**

**Comment**: Line 202: The description of how you achieved the results should be moved to the methods section.

**Response: Accepted**.

**Comment:** Line 220-221- You mean to say the participants compared it early pregnancy symptoms?

**Response**: Yes. Some female study participants compared the symptoms of nausea and vomiting induced by chemotherapy to those experienced during the early trimester of pregnancy. I have made a minor modification to the language for clarity. **Page 13, lines 300 – 302.**

**Comment**: Line 228- when using the quotes, it is good to include some description, for example, (male, age 70). It is good if you can do that for all quotes; it makes it easier to relate to what is being said.

**Response**: Accepted, and thank you for this insight. Some descriptors are added to provide context for understanding. **See all quotes.**

**Comment:** Line 248-250: It is unusual to include definitions in the results section. Consider relocating other sections if necessary.

**Response**: Accepted. The definition of quality of life is moved to the introduction part and summarized with the definition of nutrition impact symptoms. **Page 3, lines 62 – 64.**

**Comment**: For category 1.1-1.2: both are focusing treatment related symptoms: physical and psychological. I suggest considering merging them and removing sub-themes.

**Response**: Category 1.1 is “Temporal and Individual Variations,” while Category 1.2 is “Burden on Quality of Life.” These two categories represent distinct concepts. The first characterizes the onset and pattern of symptoms across individuals, while the second emphasizes the impact of these symptoms on all dimensions of life physical, psychological, social, economic, and more. Therefore, I believe they represent different entities and do not require merging.

However, if the comment refers to the codes listed under "Category 1.2. Burden Quality of Life," it is acceptable to change them to descriptive statements rather than presenting them as first-level codes or subthemes. **Page15, lines 329 – 341.**

**Comment:** For category 1.3 it is different from the rest, and I suggest you put it under a separate theme highlighting the different coping mechanisms. For example, you could discuss the use of pharmacological, dietary, and economic coping strategies. It would be helpful to expand on each of these and include relevant quotes. Additionally, I believe that Theme 2 can be integrated with this, as individual tolerance to different foods and discovering what works best for each person is a crucial aspect of coping strategies.

**Response:** Thank you for the comment. This point needs a detailed explanation.

It is well understood that generating codes, categories, and overarching themes in qualitative research is an iterative process heavily influenced by the study's objectives, the interview guide that outlines its focus, the relevant information in the transcripts, and the background or context of the participants. The primary aim of this study is to explore the nutritional challenges faced by patients undergoing chemotherapy and to suggest evidence-based recommendations for future nutrition intervention projects. Based on this understanding, we aimed to identify and document the patterns of food preferences and aversions that works for most patients, which will inform dietary counseling for these individuals.

With this perspective, we included an independent question in the interview guide: “What foods have become easier or harder for you to eat during chemotherapy, and why?” This question was the primary factor that increased the sample size to 26, as we aimed to identify common patterns of food preferences among participants. However, a substantial number of initial codes were emerged, including: apathetic toward all food items, dislike of fatty foods, preference for tubers such as potatoes, dislike of all fruits, preference for legumes (shiro), aversion to vegetables, dislike of all dairy products, preference for dairy products, preference for fruits and vegetables, , avoidance of spicy foods, and preference for spicy foods, etc. Ultimately, we concluded with the theme of “individualized food choice” to characterize the non-patterned food preferences of patients undergoing chemotherapy.

Additionally, the question regarding "food easier or harder to eat during chemotherapy” in the interview guide was not mainly intended to explore diet as a coping method for nutrition-related symptoms, although some patients mentioned the use of spicy foods (such as pepper and sauce) as coping strategies. The purpose of including this question in the interview guide was to understand the patterns of foods that are liked and disliked during chemotherapy, and to guide future interventions.

Furthermore, the codes and excerpts under the subthemes of “symptom relief methods” mainly emphasize the effectiveness of medications and psychosocial supports, with little emphasis on dietary methods. Therefore, it is important to keep “individualized food choice” as an independent theme due to its strong alignment with the objective, relevance for future nutritional interventions, and being the most frequent code that emerged during the analysis, as it is presented in **Figure 1** code summary statistics.

On the other hand, the first theme, “symptom pattern and consequences,” is intended to summarize all issues related to nutrition impact symptoms, including onset, severity, pattern, consequences, and coping methods. I understand that the wording of the theme should reflect the three subthemes under it. Based on this understanding, the theme **“symptom pattern and consequences”** has been modified to **“symptom burden and coping”** to encompass symptom relief methods in addition to the pattern and consequences. The term “burden” can refer to the severity, duration, frequency, and number of concurrent symptoms, as well as their impact on quality of life. This correction has been applied to all parts of the manuscript where themes are presented. I hope this explanation makes sense, especially as the PI conducting the interviews and data analysis. **See the schematic presentation of themes and the result section.**

**Comment:** Line 286: 291: Are you referring to their preferences in general or preference and tolerance while taking chemo?

**Response**: All the results presented in this manuscript, including the food preference, should be understood while taking chemotherapy, not in a healthier state. I hope the manuscript title also reflects this response: “Lived Experience of Nutrition Impact Symptoms among Patients Undergoing Chemotherapy in Ethiopia”.

**Comment**: Line 308-311: Doesn’t this reflect a lack of specialized nutritional support in general, instead of a misconception?

**Response: Yes.** In general, it highlights a lack of specialized nutritional support, which we refer to as the theme "unmet nutritional support needs." The nutritional misconception is a subtheme under this. The misconceptions and misinformation among patients reflect the insufficient understanding of oncologists and nurses regarding the nutritional interventions recommended for cancer patients undergoing chemotherapy. Patients seek accurate dietary information by asking doctors and nurses, searching the internet, and informally consulting their peers during treatment. However, the information they receive is often contradictory, leading to misguided behaviors.

As we employed interpretative phenomenological analysis, understanding the context of patients’ responses is essential for extracting their true meaning. The two remaining subthemes, economic inaccessibility to food and poor experience of hospital food, further reinforce the broader theme of “unmet nutritional support needs.” Some patients recognize the importance of proper nutrition for cancer care, but struggle to afford appropriate food items. Additionally, there are no nutritional supplements available in oncology pharmacies, and the hospital food does not address the specific needs of cancer patients. All of these factors contribute to the conclusion of unmet nutritional support needs within the hospital.

Thank you!
